# Predicting current and future high-risk areas for vectors and reservoirs of cutaneous leishmaniasis in Iran

**DOI:** 10.1038/s41598-023-38515-w

**Published:** 2023-07-17

**Authors:** Faramarz Bozorg-Omid, Anooshe Kafash, Reza Jafari, Amir Ahmad Akhavan, Mohammad Rahimi, Abbas Rahimi Foroushani, Fahimeh Youssefi, Mohammad Reza Shirzadi, Abbas Ostadtaghizadeh, Ahmad Ali Hanafi-Bojd

**Affiliations:** 1 Department of Vector Biology and Control, School of Public Health, Tehran University of Medical Sciences, Tehran, Iran; 2 Zoonoses Research Center, Tehran University of Medical Sciences, Tehran, Iran; 3 School of Public Health, Esfahan Health Research Station, Tehran University of Medical Sciences, Tehran, Iran; 4 Department of Combat Desertification, Faculty of Desert Studies, Semnan University, Semnan, Iran; 5 Department of Epidemiology and Biostatistics, School of Public Health, Tehran University of Medical Sciences, Tehran, Iran; 6 Department of Photogrammetry and Remote Sensing, Faculty of Geodesy and Geomatics Engineering, K. N. Toosi University of Technology, Tehran, Iran; 7 Center for Research of Endemic Parasites of Iran, Tehran University of Medical Sciences, Tehran, Iran; 8 Center for Communicable Diseases Control, Ministry of Health and Medical Education, Tehran, Iran; 9 Department of Health in Emergencies and Disasters, School of Public Health, Tehran University of Medical Sciences, Tehran, Iran

**Keywords:** Ecological modelling, Infectious diseases

## Abstract

Climate change will affect the distribution of species in the future. To determine the vulnerable areas relating to CL in Iran, we applied two models, MaxEnt and RF, for the projection of the future distribution of the main vectors and reservoirs of CL. The results of the models were compared in terms of performance, species distribution maps, and the gain, loss, and stable areas. The models provided a reasonable estimate of species distribution. The results showed that the Northern and Southern counties of Iran, which currently do not have a high incidence of CL may witness new foci in the future. The Western, and Southwestern regions of the Country, which currently have high habitat suitability for the presence of some vectors and reservoirs, will probably significantly decrease in the future. Furthermore, the most stable areas are for *T. indica* and *M. hurrianae* in the future. So that, this species may remain a major reservoir in areas that are present under current conditions. With more local studies in the field of identifying vulnerable areas to CL, it can be suggested that the national CL control guidelines should be revised to include a section as a climate change adaptation plan.

## Introduction

Earth's climate was not static in the past and will not be in the future^1^. Iran, in particular, is highly vulnerable to the impact of the undeniable phenomenon of climate change, with estimates projecting a noteworthy increase between 1.12 and 7.87 °C in temperature and a decline by 35% in precipitation over the next decade^2^. Such climatic alterations have significant effects on the distribution patterns of various species, including vectors and reservoirs of diseases^3^, and have a vital role to play in the epidemiology of vector-borne diseases (VBDs)^4^.

Cutaneous leishmaniasis (CL) is the most prone epidemic of VBDs in Iran and the world and is currently known as the most important disease in terms of the high incidence and occurrence of multiple epidemics in Iran^5^. Iran is one of the six countries in which more than 95% of CL incidents occur^6^. In the past decade, several epidemics of anthroponotic-CL (ACL) and zoonotic-CL (ZCL) have been reported on different scales^7–12^, which can be caused by the exposure of the non-immune population to the vectors (*Phlebotomus papatasi* and *Phlebotomus sergenti*) and the expansion of agriculture in rural areas, which increases the population of reservoirs (*Rhombomys opimus*,* Meriones libycus*,* Tatera indica*, and *Meriones hurrianae*) and changes their distribution pattern^13^.

Recent studies have predicted that climate change will also affect on the transmission of VBDs^14^. A body of evidence-based studies has proven that climate change has affected the transmission of leishmaniasis in different geographic areas worldwide, prompting researchers to stress the need for making well-informed future predictions about the potential expansion or shrinkage of vectors and reservoir species^15–18^. Besides this, several studies have accentuated this argument with alter the distribution of vectors and reservoirs of several major diseases in Iran^19, 20^. Therefore, the disease requires important surveillance and proactive control measures, requiring progress in surveillance networking. As a crucial step toward this objective, decision-makers must plan and predict the effects of climate change on the distribution of CL vectors and reservoirs in the coming decades, making sustainable and informed decisions to mitigate the potential risks^16, 18, 21^.

Species distribution models (SDMs), also known as Ecological Niche Models (ENMs), are very practical tools for predicting the impacts of climate change on species^22^. These models can be used to understand the responses of vectors and reservoirs to future climate changes^19, 23–25^. These models use occurrence records of insects/plants and environmental data to predict their habitats with a high probability of the presence of the target species^26–28^. In a retrospective review study, more than 35 modeling methods were identified for generating SDMs^29^. Given the abundance of available models, it is uncertain which has the best predictive performance. In other words, each one has its advantages and disadvantages^22^. As a result, no existing model can accurately predict the distribution of all species. From 2006 onwards, researchers have highlighted the importance of comparing SDMs with more than one modeling method^29^, and it is recommended that it is better to use several models simultaneously, which makes it possible to better decide which one fits best and has the best function on the distribution of species or to identify areas at risk^27, 29^. The most common combination performed in the world is related to the use of the maximum entropy (MaxEnt) model with other SDMs (generalized linear model—GLM, random forest—RF, generalized boosting model—GBM, and others)^29^.

While a couple of studies based on representative concentration pathway (RCP) scenarios have been conducted in the field of predicting risks of various VBDs in Iran^19, 20, 30, 31^, no study has yet tested shared socioeconomic pathways (SSPs) scenarios. However, models have seldom been compared to assess the effects of climate change on VBDs. We conducted this study based on a set of SSP scenarios with the approach of two-SDM to compare models’ performance simultaneously in predicting changes in the distribution pattern of the main CL vectors and reservoirs in whole Iran territories by 2050, and to explore CL transmission risk at the country-level to provide scientific evidence for CL management in the pre-emergency phase.

## Results

### Current and future distribution of CL vectors

**Figure 1:**
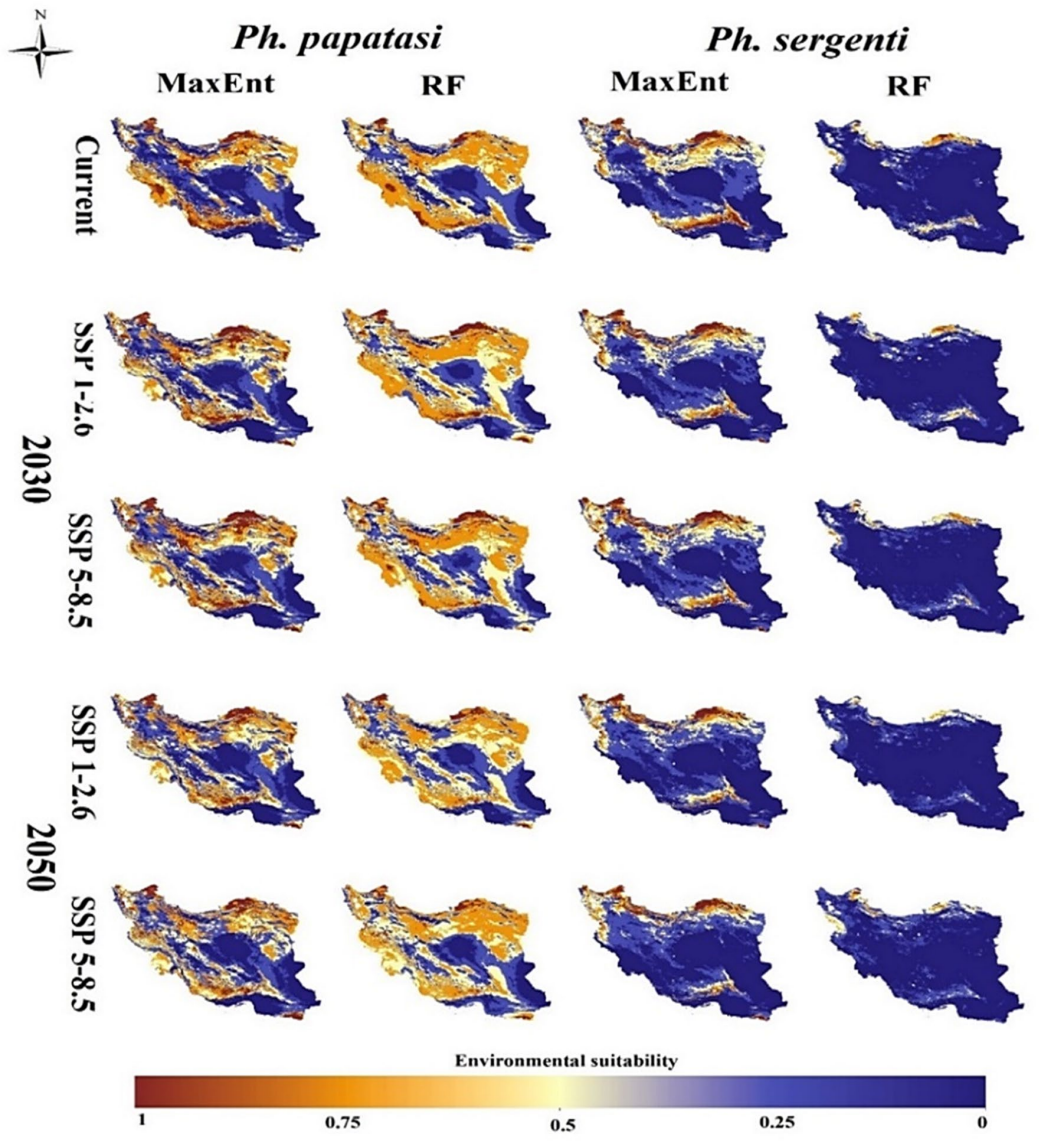
Current and future (2030s and 2050s) distribution models (MaxEnt and RF) of the two cutaneous leishmaniasis vectors (*Phlebotomus papatasi* and *Phlebotomus sergenti*) in Iran.

Two models showed that the areas favorable to *Ph. papatasi* will change in the future (comparison of the distributions according to the periods and according to the models). Under the current conditions, *Ph. papatasi* is located in the west (Ilam and Khuzestan provinces), southwest (Bushehr Province), south (Fars and Kerman provinces), and a small part of the southeast (South of Sistan-Baluchistan Province (of the country. However, the species is distributed from the center (Qom, Tehran, Semnan, and Esfahan provinces) to the east (Khorassan-Razavi Province), north (Golestan and Khorassan-Shomali provinces), and even a part of the northwest (Ardabil Province). Overall distribution models developed based on MaxEnt and RF are similar but have a different probability of presence. The RF model showed the possibility of this species being present a wider range of areas. For example, in the west, parts of Kurdistan and Lorestan Provinces have also estimated suitable habitats for the presence of this species in the current conditions, and in the center and east, which are distributed in a wider range in Semnan and Khorassan-Razavi Provinces, respectively (Fig. 1).

Both models showed that the future suitable area of ​​*Ph. papatasi* will change under different climate scenarios and in both periods. Modeling based on MaxEnt under both scenarios related to the period 2030 shows that the probability of the presence of this species may shrink dramatically in the Ilam and Khuzestan (west), Bushehr (southwest), and Fars (south) provinces. Instead, this model has shown some provinces located in the west (Kurdistan), northwest (Azerbaijan-Gharbi), center (Alborz, Qazvin, and north of Semnan), east (Khorassan-Razavi), and southeast (Sistan-Baluchistan) as hotspots for the *Ph. papatasi* species and suitable habitat areas will probably increase. The two scenarios related to the period of 2050 also predicted the same as the period of 2030, but the only difference between that is the increase in the probability of the presence of this species in the south of Sistan-Baluchistan Province (Chabahar and Konarak Counties) will be higher than that in the period of 2030. On the other hand, although the presence of this species in Tehran, Qom, Esfahan, and part of Semnan provinces will increase according to different scenarios in the 2030s, in 2050s, we will probably see a decrease in the presence of this species in those Provinces. The RF model also predicted the same situation for this species, but according to the predictions of the RF model, in some Provinces such as Mazandaran and Gilan (in the north), the presence of this species will probably increase in the future (Fig. 1).

**Figure 2:**
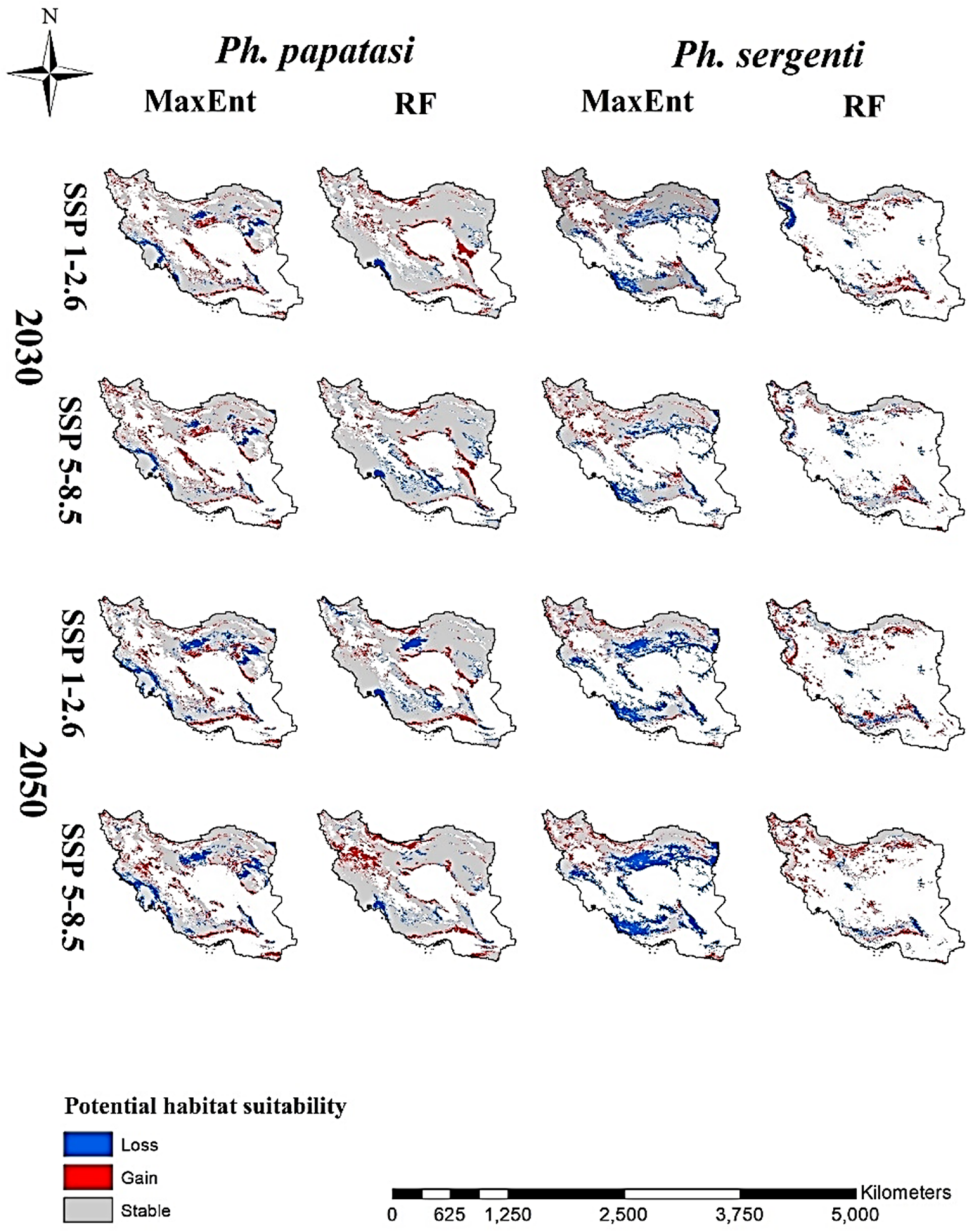
Gain, loss and stable maps for the two cutaneous leishmaniasis vectors (*Phlebotomus papatasi* and *Phlebotomus sergenti*) under two climate change scenarios in the 2030s and 2050s in Iran.

According to both models, in the most optimistic scenarios (SSP1-2.6) until 2030, the calculated gain area for *Ph. papatasi* was greater than the loss. This situation is also true in both models for the 2050s-SSP5-8.5 scenario. In contrast, the 2050s-SSP1-2.6 scenario estimated the area of loss to be greater than gain. The only difference between the two models is related to the 2030s-SSP5-8.5 scenario in which the RF model, contrary to MaxEnt, shows a greater area of loss than gain. The maximum gain areas according to the MaxEnt and RF models were estimated to be 123,210 and 128,444 km^2^, respectively, which was predicted by the 2050s-SSP5-8.5 scenario for both models. The maximum loss area was predicted in the 2050s, which was predicted for the MaxEnt and RF models under scenario SSP5-8.5 and SSP1-2.6, equivalent to 122,327 and 88,411 km^2^, respectively. On the other hand, the minimum gain and loss area was predicted in the 2030s, where the MaxEnt model predicted the gain and loss area as 106,805 and 63,237 km^2^, and the RF model has predicted 71,699 and 31,925 km^2^, respectively (Figs. 2, 5, Table 1).

**Table 1:** Species distribution ranges of cutaneous leishmaniasis vectors and reservoirs species in different periods under different scenarios, Iran.

Periods	Scenarios	Models	*Ph. papatasi*			*Ph. sergenti*			*R. opimus*			*M. libycus*			*T. indica*			*M. hurrianae*		
			L	G	S	L	G	S	L	G	S	L	G	S	L	G	S	L	G	S
2030	SSP1-2.6	MaxEnt	72,370	106,805	522,134	135,226	63,475	480,255	283,854	42,264	229,054	231,488	61,299	395,886	36,177	37,976	310,068	650	6649	49,756
		RF	31,925	114,603	783,087	57,202	79,164	166,216	38,808	126,436	143,242	88,626	200,737	193,867	34,020	18,717	435,424	10,527	295	39,620
	SSP5-8.5	MaxEnt	63,237	119,326	531,084	120,273	75,226	494,008	286,410	42,916	226,423	232,227	65,700	395,092	37,802	39,321	309,642	502	8074	49,899
		RF	81,970	71,699	731,888	63,909	63,909	159,380	39,929	215,047	142,453	85,262	167,233	197,239	78,829	18,015	391,272	3209	2327	46,900
2050	SSP1-2.6	MaxEnt	116,641	110,458	477,858	214,369	49,362	399,802	333,284	39,136	179,817	338,612	63,412	288,765	42,501	48,207	304,185	1571	8180	48,835
		RF	88,411	83,935	725,406	58,645	97,552	164,632	47,963	346,251	131,741	105,649	219,190	176,407	93,451	17,616	375,757	1458	6410	48,690
	SSP5-8.5	MaxEnt	122,327	123,210	472,177	264,115	63,893	350,420	386,074	41,549	179,817	417,690	75,329	209,759	42,455	56,138	304,471	1450	16,671	48,956
		RF	50,923	128,444	764,023	41,646	112,221	181,655	56,272	391,760	126,079	66,992	613,276	215,266	81,519	19,526	387,978	3209	2327	46,900

*L* loss (km^2^), *G* gain (km^2^), *S* stable (km^2^).

*Phlebotomus sergenti* has a more limited distribution and the suitable areas for it are mainly located in the south (south of Fars, and Kerman provinces), north (Golestan and Khorassan-Shomali provinces) to the northwest (Ardabil and Gilan provinces) areas and also in parts of Tehran and Bushehr provinces located in the center and southwest of the country, respectively. In the MaxEnt model, the presence probability of this species in all areas was higher more than in the RF model (Fig. 1).

According to the MaxEnt model and under the 2030s-SSP1-2.6 scenario, the probability of the presence of *Ph. sergenti* significantly decreased in the south and southwest (Fars, Kerman, and Bushehr provinces) over time and under different climate scenarios. It can only be said that the presence of this species will probably increase in the center (Tehran Province), south of Sistan-Baluchistan Province (Chabahar County), the northwestern provinces (Gilan, Ardabil, and north of Azerbaijan-Sharghi and Azerbaijan-Gharbi). Changes in the presence of *Ph. sergenti* under both scenarios in the 2050s were predicted to be similar to those in the 2030s. Thus that the 2050s scenario predicted a significant decrease in the presence of this species in Kerman, Fars, and Bushehr provinces and an increase in Chabahar County. There is a difference in the prediction of the two models in the 2030s-SSP1-2.6 scenario; thus the RF model shows that a significant decrease in attendance can be seen in the west (Ilam and Kurdistan provinces). However, Chabahar County will not be a suitable area for *Ph. sergenti*. However, in other scenarios it can be a suitable habitat for the species (Fig. 1).

Under each future climate change scenario and for both periods, the MaxEnt model overestimated the loss area more than the gain area did. This case is completely the opposite of the RF model; therefore, the RF model estimated the gain area more than the loss area. In all scenarios, the calculated gain area value for *Ph. sergenti* in the RF model was higher than that in the MaxEnt model, but the loss area calculated by the MaxEnt model was greater than that of the RF model. The MaxEnt model showed that the maximum gain and loss areas were 75,226 and 264,115 km^2^ under scenarios the 2030s-SSP5-8.5 and the 2050s-SSP5-8.5, respectively. However, according to this model, the minimum gain and loss area was predicted under the scenarios 2050s-SSP1-2.6 and 2030s-SSP5-8.5 as 49,362 and 120,273 km^2^. However, the RF model predicted the maximum gain and loss area for *Ph. sergenti* under the 2050s-SSP5-8.5 and the 2030s-SSP5-8.5 scenarios as 112,221 and 59,549 km^2^, and the minimum gain and loss area predicted by this model were 63,909 and 41,646 km^2^ under the 2030s-SSP5-8.5 and the 2050s-SSP5-8.5 scenarios, respectively (Figs. 2, 5, Table 1).

It determines, according to the models, the most important environmental variables to predict the air distribution of *Ph. papatasi* and *Ph. sergenti* were Bio3–Bio12 and Bio1–Bio15, respectively (Table 2).

**Table 2:** Models performance and the most important environmental variables for cutaneous leishmaniasis vector and reservoirs species based on the MaxEnt and RF models.

Species	*Ph. papatasi*	*Ph. sergenti*	*R. opimus*	*M. libycus*	*T. indica*	*M. hurrianae*
AUC (MaxEnt-RF)	0.798–0.823	0.804–0.937	0.832–0.935	0.778–0.923	0.878–0.879	0.986–0.978
Variables importance (MaxEnt-RF)	Bio3-Bio12	Bio1-Bio15	Bio1-Bio15	Bio1-ALT	ALT-Bio1	Bio4-Bio1

### Current and future distribution of CL reservoir hosts

**Figure 3:**
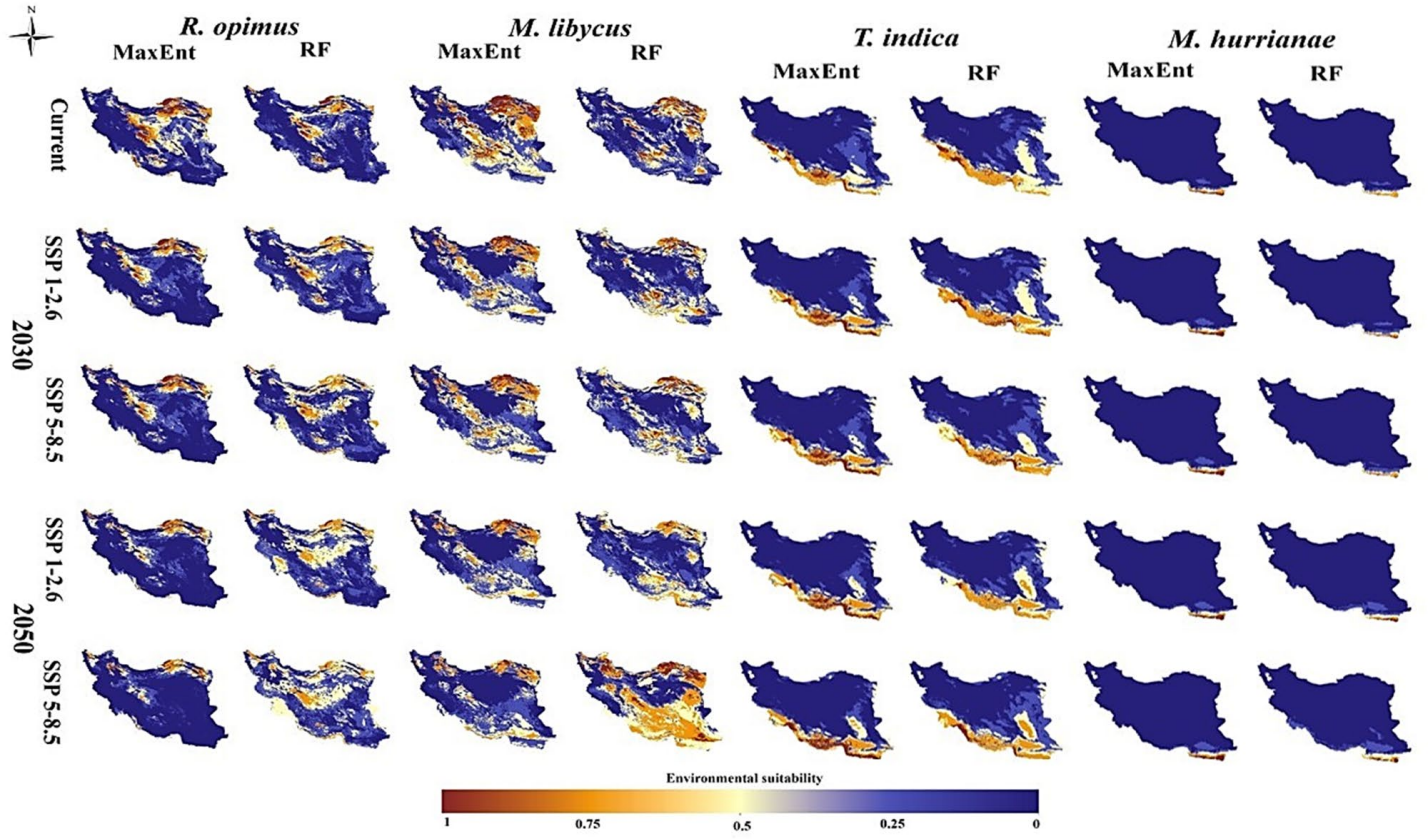
Current and future (the 2030s and 2050s) distribution models (MaxEnt and RF) of the four zoonotic cutaneous leishmaniasis reservoirs (*Rhombomys opimus*, *Meriones libycus*, *Tatera indica*, and *Meriones hurrianae*) in Iran.

According to both models, *R. opimus* is mostly present in the central region (that is Esfahan and Qom provinces), northern (Golestan, Khorasan-Shomali, and Semnan provinces), east (Khorasan-Razavi Province), and south (Fars Province) regions under current climatic conditions. There were minor differences between the two models in predicting the presence of this species, for example; the RF model did not show the southwest (Bushehr Province) in the current favorable conditions for the presence of this species (Fig. 3).

Description of the reservoir distribution according to the two models which showed a decrease in the *R. opimus* species in the country in 2030s. The most significant decrease is related to the central area (Esfahan and Qom provinces), north of Semnan and northeast of Khorassan-Razavi, especially according to the 2050s-SSP5-8.5 scenario. In addition, the RF model predicts an increase in the probability of the presence of this species in the period of 2030s-SSP5-8.5 in the southern regions (south of Hormozgan Province). The models also predicted that the northern parts of the country such as Golestan Province will continue to be a suitable habitat for the presence of *R. opimus* in the future under different climate scenarios (Fig. 3).

**Figure 4:**
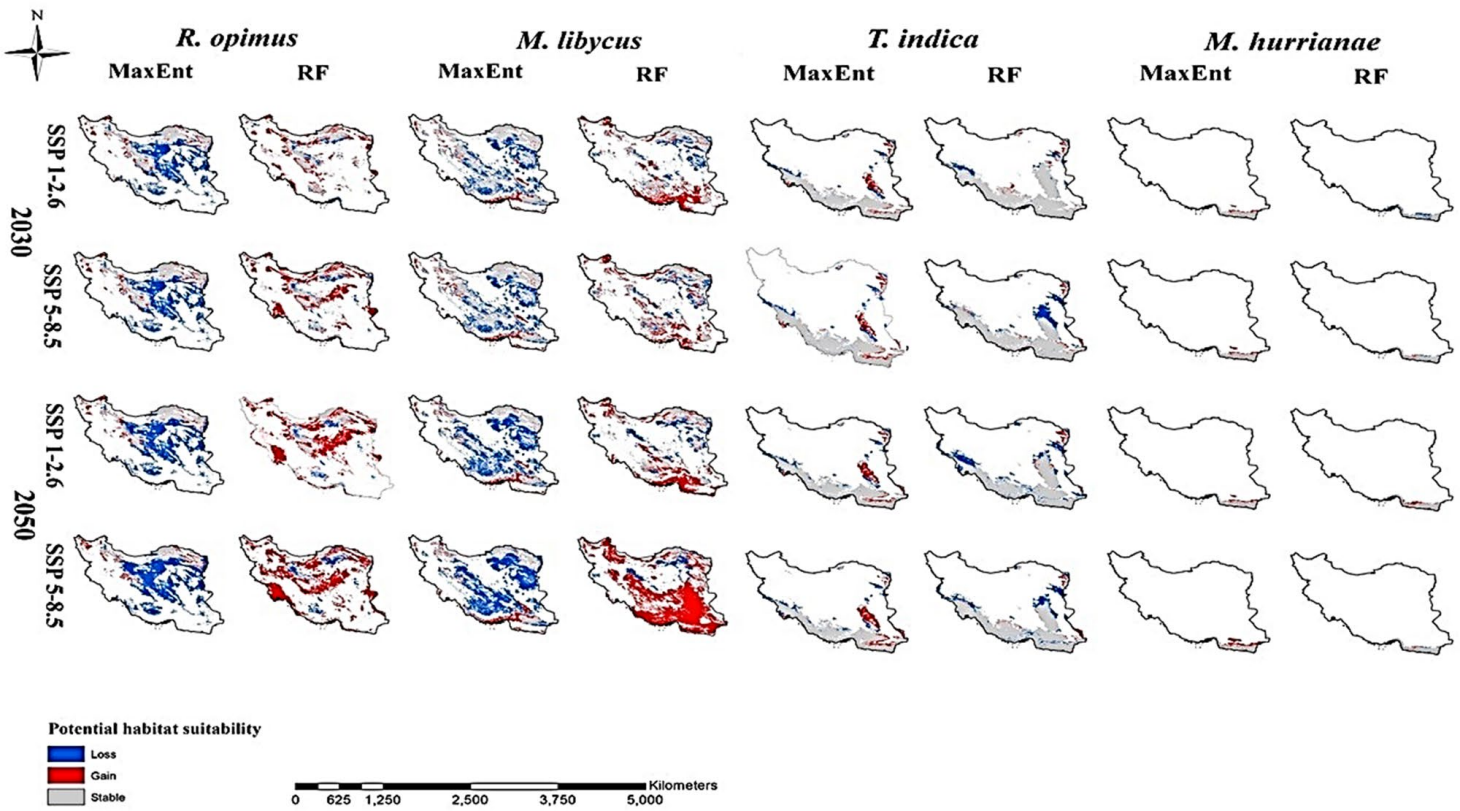
Gain, loss and stable maps for the four zoonotic cutaneous leishmaniasis reservoirs (*Rhombomys opimus*, *Meriones libycus*, *Tatera indica*, and *Meriones hurrianae*) under two climate change scenarios in the 2030s and 2050s in Iran.

**Figure 5:**
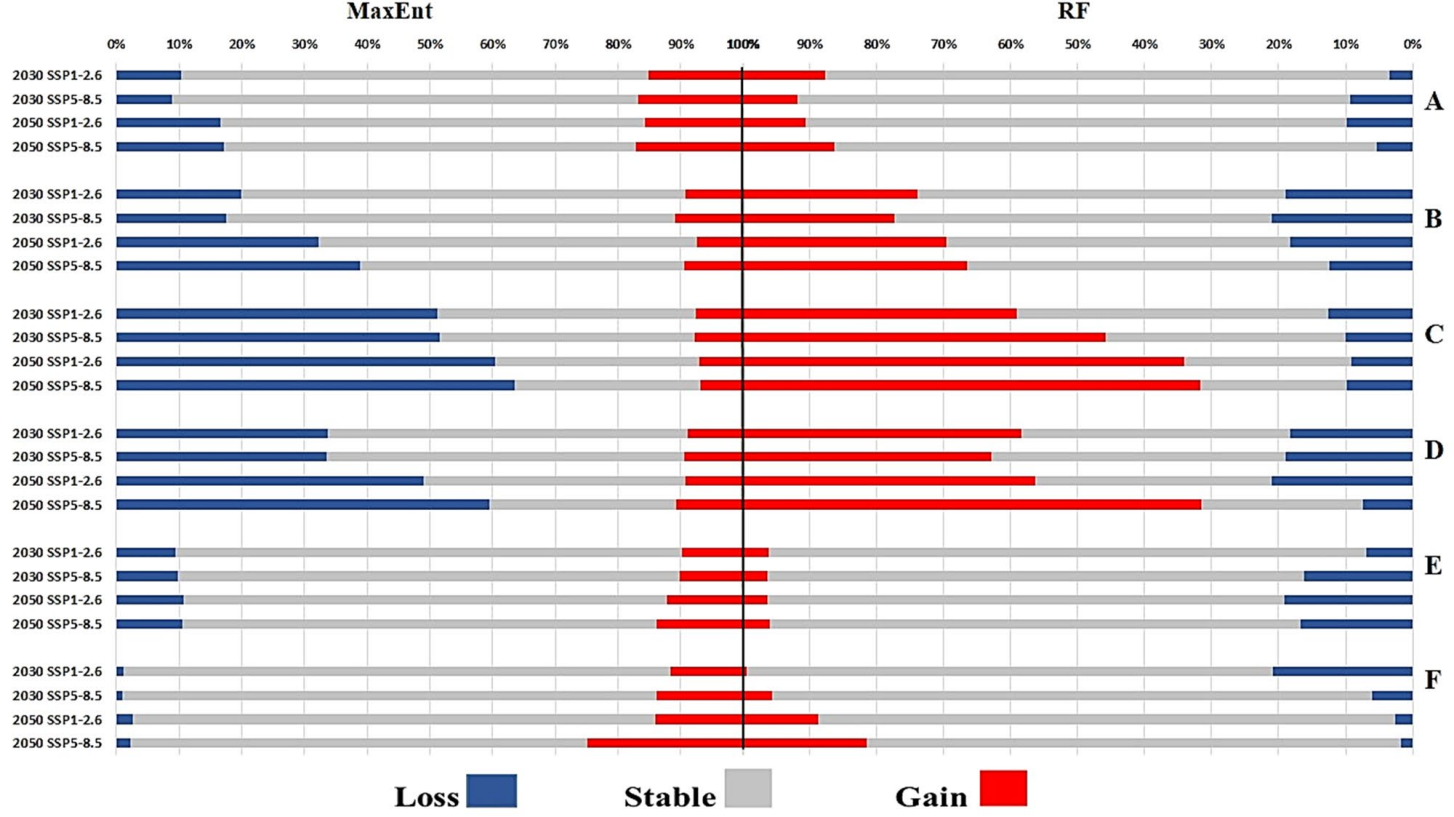
Percentage gain, loss and stable of the distribution area of cutaneous leishmaniasis vectors (**A**: *Phlebotomus papatasi*, **B**: *Phlebotomus sergenti*) and reservoirs (**C**: *Rhombomys opimus*, **D**: *Meriones libycus*, **E**: *Tatera indica*, and **F**: *Meriones hurrianae*) species in different periods under different scenarios, Iran.

Unlike the RF model, the MaxEnt model estimated that the loss area for *R. opimus* was larger than the gain area for all scenarios and periods. The RF model under the 2050s-SSP5-8.5 scenario showed that maximum gain and loss occurred, with area of 391,760 and 56,272 km^2^, respectively. This scenario also showed the maximum loss (386,074 km^2^) in the MaxEnt model. The RF model also showed the minimum gain and loss area under the 2030s-SSP1-2.6 scenario and their area were estimated to be 126,436 and 38,808 km^2^, respectively (Figs. 4, 5, Table 1).

The MaxEnt and RF models predicted the presence of *M. libycus* under the current conditions of the north-to-east (Golestan, Semnan, Khorassan-Shomali, Khorassan-Razavi and Khorassan-Jonoobi provinces), center (Esfahan and Qom provinces), and the south (Fars and Kerman provinces). Compared with the RF model, the MaxEnt model has predicted more territory for the distribution of this species. The models predicted that in the future their current areas will not be suitable for the presence of this species and a significant decrease will occur especially in the 2050s in the north, the center (especially Qom and Esfahan provinces), and the northern part of Fars and Kerman provinces. However, according to both models, areas located south of Kerman, Hormozgan, and Sistan-Baluchistan provinces are expacted to become more favorable habitats for this species in the future (Fig. 3).

Unlike the RF model, the MaxEnt model estimated that the loss area for *M. libycus* was larger than the gain area in any scenarios and in both periods. According to the MaxEnt model, the maximum gain and loss area for *M. libycus* was shown by the 2050s-SSP5-8.5 scenario, whose areas were estimated to be 75,329 and 417,690 km^2^, respectively. In addition, this model showed the minimum gain and loss area under the 2030s-SSP1-2.6 scenario, which were predicted to be 61,299 and 231,488 km^2^, respectively. In contrast, the maximum and minimum RF models estimated the area of gain as 613,276 and 167,233 km^2^, which were predicted under the 2050s-SSP5-8.5 and the 2030s-SSP5-8.5, respectively. In addition, the maximum and minimum loss areas calculated by this model under the 2050s-SSP1-2.6 and 2050s-SSP5-8.5 scenarios were approximately 105,649 and 66,992 km^2^, respectively (Figs. 4, 5, Table 1).

Both models show a similar pattern for the presence of *T. indica* under current conditions; therefore, the favorable areas for the presence of this species are located in the west (Lorestan and Khuzestan provinces), southwest (Bushehr Province), south (Hormozgan Province, south of Kerman and Fars provinces) and southeast (south of Sistan-Baluchistan Province) (Fig. 3).

In future climatic conditions, two models predicted a significant decrease in the probability of the presence of this species in the Provinces located in the west (Ilam and Khuzestan). On the other hand, it is expected that the presence of this species in Sistan-Baluchistan Province (Konarak, Chabahar, Iranshahr, and Delgan Counties) and the south of Kerman Province (Rodbar-Jonoob and Ghaleganj Counties) will increase significantly. These changes are better observed in the 2050s-SSP126 scenario. The only difference between the two models is related to the 2050s-SSP585 scenario, in which the RF model predicts that the probability of this species will increase in the west (Khuzestan Province), unlike MaxEnt (Fig. 3).

The MaxEnt model estimated the gain area more than the loss area in each scenario for both the 2030s and the 2050s for *T. indica*. This situation is exactly the opposite of that of the predicted RF model. According to the MaxEnt model, the maximum gain and loss area is predicted as 2050s-SSP585 scenario, which is equal to 56,138 and 42,455 square kilometers (km^2^), respectively. Furthermore, the minimum gain and loss area are related to scenario 2030s-SSP126, which were estimated at 37,976 and 36,177 km^2^, respectively. The maximum gain and loss area predicted by the RF model are related to scenarios 2050s-SSP585 and 2050s-SSP126, which were estimated at 19,526 and 93,451 km^2^, respectively. On the other hand, this model estimated the minimum gain and loss area to be 17,616 and 34,020 km^2^, which correspond to the 2050s-SSP126 and the 2030s-SSP126 scenarios, respectively (Figs. 4, 5, Table 1).

Under the current climatic conditions, in general, both models predicted the presence of *M. hurrianae* in the same way, and the suitable areas for this species were limited to the south (Hormozgan Province) and southeast (south of Sistan-Baluchistan Province) of the country. The RF model predicted a larger area of these areas for the presence of this species, and almost all of Hormozgan Province was favorable, but the MaxEnt model showed only the counties of Jask and Sirik as favorable areas (Fig. 3).

Compared to the currently suitable area, except for the 2030s-SSP126 scenario, the predictions of the two models under all scenarios in the 2030s and the 2050s shows that the potentially suitable area will expand towards higher areas (Nikshahr, Ghassreghand, Sarbaz, and Bashagard counties). On the other hand, only in the 2030s-SSP126 scenario will the habitat desirability of this species decrease in Bashagard and Nikshahr. These changes were also observed in the RF model (Fig. 3).

The MaxEnt model has estimated under both scenarios of the 2030s and the 2050s that the area of gain was greater than the loss for *M. hurrianae*. This situation is true for the RF model in the 2050s. The only difference between the two models is related to the 2030s scenario in which the RF model, contrary to MaxEnt, shows a greater area of loss than gain. The MaxEnt model predicted the maximum area of gain and loss for *M. hurrianae* according to the scenarios 2050s-SSP585 and 2050s-SSP126, and their values were 16,671 and 1571 km^2^, respectively. In addition, this situation for the RF model is equal to 11,732 and 10,527 km^2^, which is the result of the prediction of 2050s-SSP585 and 2030s-SSP126 scenarios, respectively. On the other hand, both MaxEnt and RF models predicted the minimum gain area under the 2030s-SSP126 scenario which was estimated at 6649 and 295 km^2^, respectively. In addition, the minimum loss area was predicted as 502 km^2^ by the MaxEnt model under the 2030s-SSP585 scenario and 1139 km^2^ useing the RF model under the 2050s-SSP585 scenario. The MaxEnt model showed that the total calculated gain area was greater than the loss area for *M. hurrianae* according to any scenarios in the 2030s. This situation is also true in the RF model only for 2050s-SSP126 scenario (Figs. 4, 5, Table 1).

According to MaxEnt the high-importance environmental variables for predicting the potentially suitable area of *R. opimus*, *M. libycus*, *T. indica*, and *M. hurrianae* were Bio1, Bio1, ALT, and Bio4, respectively. The most important variables shaping the distribution of those species in the RF models were Bio15, ALT, Bio1, and Bio1, respectively (Table 2).

### Model performance

The results showed that 2 models perform well in relation to the values of the area under the curve. In the majority of cases these values were higher for the RF model compared to those of the MaxEnt model, showing a better predictive performance of the RF model (Table 2).

### Field validation of the species distribution model

Presence/absence results for *Ph. papatasi*, *R. opimus* and *M. libycus* showed that the models made a good prediction, locations where the probability of presence was > 60%, all three species were captured and identified.

Although the probability of the presence of species in the Araghavanieh area was estimated to be above 60%, no species were caught due to land use changes in that area. On the other hand, in areas where there were less than a 20% probability of species presence, no species were caught (Table 3).

**Table 3:** Results of field validation of species distribution models along with geographical coordinates of the selected districts, Esfahan Province of Iran, 2022.

	County	Location	District	Geographical coordinates	*Ph. papatasi*	*R. opimus*	*M. libycus*
Districts with a presence probability > 60%	Shahin Shahr	1	Chah Naji	51.609 N 32.8172989 E	Yes	No	Yes
	Borkhar	2	Jurabi	51.751 N 32.8087997 E	Yes	No	Yes
	Ardestan	3	Nusrat Abad	52.188 N 33.6231995 E	Yes	No	Yes
	Natanz	4	Moazi Abad	52.051 N 33.6869011 E	Yes	Yes	No
	Esfahan	5	Fassaran	51.999 N 32.5620003 E	Yes	Yes	No
		6	Araghavanieh	51.773 N 32.6423988 E	No	No	No
Districts with a presence probability < 20%	Esfahan	7	Mir Lotfullah	52.795 N 32.7434006 E	No	No	No
		8	Tenijan	52.447 N 32.8525009 E	No	No	No
		9	Mazraeh mushu	52.337 N 32.9636002 E	No	No	No
	Shahin Shahr	10	Imamzadeh Yusuf	51.462 N 33.4449005 E	No	No	No
		11	Zere pol	51.562 N 33.4770012 E	No	No	No

*Yes* presence, *No* absence.

## Discussion

Dropping the public health burden of VBDs primarily depends on the prospective determination of vulnerable areas caused by the presence of vectors and reservoirs^32^. Because of the uncertainty of choosing the appropriate technique to identify suitable habitats for species, the use of different types of models can help make predictions close to reality^33^. Use here of machine learning, MaxEnt, and Rf models to compare their performance in predicting suitable habitats for 6 vectors and reservoirs of cutaneous leishmaniasis in Iran. Studies have shown that machine learning methods are more powerful than traditional regression-based algorithms^34^. MaxEnt and RF models are widely regarded as machine learning algorithms for species distribution mapping^35, 36^. In recent years, a growing literature has been created to compare a couple of the modeling methods’performance to determine the best model^29, 35–37^. It is important to note that the performance of an SDM is evaluated only for its designed purpose^38^. In other words, the performance of a given model varies depending on the quality of the response variable, predictor variables, model building, and model evaluation aspects^39^.

The validity of the model in our study for prediction under current conditions was first tested in comparison with data collected independently from areas not yet surveyed for species presence to avoid sampling bias^33, 40^. As *Anderson *et al. pointed out, bias in sampling efforts can potentially distort the performance of models^41^. Field surveys for catching *Ph. papatasi*, *R. opimus*, and *M. libycus* in settings with a probability of presence above 60% and below 20% showed that both models have correctly predicted the presence and absence areas of those species under current climate conditions. Previous studies have also confirmed the acceptable performance of models through field evaluations^42^. In the first step, these findings indicate that modeling outputs can be trusted for the projection of future conditions.

Next, we discuss the results of evaluating the ability to predict habitat where the mapped predictions were quantitatively and visually assessed. The AUC is considered the best evaluation of predictive power and has been extensively used in SDMs^37, 43^. AUC indicated that the two models were able to perform acceptable prediction (AUC ≥ 0.75) for all species distributions, but with little variation between AUC values. In other words, the AUC was higher in the RF model, indicating better performance in the RF model. Our result agrees well with those of other studies and confirmed the performance of RF rather than MaxEnt in terms of AUC index^35–37^. Our results do not explain why one model performs better than the other; however, possibly the superior predictive performance of a particular model may be due to methodological advances in machine learning, improved mathematical modeling techniques, and more powerful statistical tools^43^. Many other factors, such as sample size, spatial scale, selection of environmental variables, and selection method for pseudo/absence data can affect predictive performance^44, 45^. Therefore, methodological improvements may reduce potential problems in modeling and increase their accuracy.

In our study, the MaxEnt model tended to predict high values across any area, whereas the RF model predicted gradations in suitability more accurately. Both the MaxEnt and RF models showed that under any climate scenario, the distribution of both of CL vectors will change in the future. The projected maps show that by 2050, compared to the current climatic conditions, areas in the northwest (from Gilan to Azerbaijan-Gharbi Provinces) will find suitable habitats for both species in Iran. Consistent with our study, in Iran and even Europe expansion to high altitudes is predicted in the modeled distribution areas of sand fly species^19, 46, 47^. Although the disease in some of these areas has not yet been reported or has a low incidence, these areas should be considered for field studies more than in the past to prevent the creation of possible new CL foci in Iran. In addition, on the other side of the country, in the southeast, mainly Chabahar towards Konarak County, there will be more suitable habitats due to climate change caused by the presence of these species. Moreover, the presence of two vectors in the western, central, and southern parts of the country, which are important foci of CL disease^5^, may decrease significantly. In other words, reducing the presence of *Ph. papatasi* in the Provinces of Ilam, Khuzestan, Bushehr, Qom, North of Esfahan, and South of Fars provinces as well as, reducing the presence of *Ph. sergenti* in Kerman and Fars provinces will probably change the pattern of the ZCL and ACL in the country, respectively.

The distribution of the four studied gerbils as the main reservoir hosts of the ZCL in the country is also expected to be affected by climate change^19^. The results of our modeling using MaxEnt and RF models for *R. opimus* showed that suitable habitat for the great gerbil will be almost the same as the current situation, although the area of the hot spots will probably be a significant decrease in suitable habitats in the country, especially in Esfahan Province. We expect to see this situation more so in the 2050s. Our findings are consistent with a recent prediction of the potential distribution pattern of *R. opimus* that the area of suitable habitat for this species gradually decreases not only in Iran but also in China, Afghanistan, and Turkmenistan^18^, reflecting the comparability of our findings. Additionally, *M. libycus* is the main reservoir of ZCL in the absence of *R. opimus*^19^. Both models predicted that the presence of *M. libycus* will significantly decrease in the future in the foci of the disease, namely Qom, Esfahan, north of Fars provinces, and even some Provinces located in the north of the country. On the other hand, the southern regions will be favorable for this species. The maps of our study show that *M. hurrianae* is found in two Provinces of Iran located in the southeast, which is reported as the reservoir of ZCL in that region^48^. Most changes in distribution are related to the increase in the probability of the presence of this species in some counties of the Sistan-Baluchistan (Ghassreghand, Sarbaz, and Nikshahr) and Hormozgan (Bashagard). On the other hand, habitat suitability for *T. indica* increases in Sitan-Baluchistan and south of Kerman (Rodbar-Jonoob and Ghaleganj). Considering that *M. hurrianae* is an oriental species, it has a limited role in the transmission cycle of ZCL in areas where *T. indica* has a higher density.

To date, the state of CL in Iran has shown that provinces such as Fars, Esfahan, Khuzestan, and Ilam always have the highest incidences^5, 19, 49^, and the highest prevalence was reported in provinces located in arid regions^50^. The spatial overlap of vectors/reservoirs with CL in these provinces was determined using our studied models. The main goal of vectors and reservoirs spatial analysis is to understand the current epidemiological situation, and the future burden of these diseases^50^. Studies have concluded that leishmaniasis is a climate-sensitive disease, and changes in the environment and distribution of vectors/reservoirs can impact the epidemiology of the disease^19, 51^. It is predicted that reducing the growth of habitat suitability of vectors and reservoirs in the current foci of CL (such as Ilam and Khuzestan) and increasing their presence in the south can push the disease pattern to the Southern Provinces. Therefore, as a general result of this study, it can be stated that a unified strategy cannot be applied to the whole country, and control programs should be used according to the climate change in each region. Relevant departments and decision-makers should pay close attention to the risk of ZCL transmission, especially in Provinces with active foci of the disease. To prevent the occurrence of disease and epidemics in these areas, it is necessary to coordinate between departments to exchange information before the implementation of large-scale construction projects (such as the construction of residential areas, sports centers, tourism, and agricultural development)^52^.

On extensive spatial (e.g. a country) and temporal (e.g. up to the 2050s) scales, species distribution is mainly limited by abiotic factors^53^. Overall, the importance of the variable varies according to algorithm^54^. The current findings from MaxEnt indicate the main role of temperature (Bio3 and Bio1) in determining the presence of *Ph. papatasi* and *Ph. sergenti* in Iran, respectively. This is probably because MaxEnt predicted more distribution changes toward warmer (southern) regions. In contrast, RF has predicted both vectors to the Provinces with higher latitudes and has placed precipitation (Bio 12 and Bio15) as the most important factor in the distribution of *Ph. papatasi* and *Ph. sergenti*, respectively. Other modelling studies on these vector species indicated that temperature^55, 56^, and precipitation^57^ when used in isolation had the greatest effect on the model in different geographical areas. Furthermore, the results of modeling using two “MaxEnt” and “RF” models for *R. opimus*, *M. libycus*, *T. indica*, and *M. hurrianae* species showed that “Bio1, Bio1, ALT and Bio4” and “Bio15, ALT, Bio1, and Bio1”, are the most important in shaping the distribution of reservoirs, respectively. Several studies that were conducted in Iran were mainly based on the MaxEnt model, and their results just were in agreement with the results of the MaxEnt model in our study^19, 55, 58^.

Although our results are in disagreement with a couple of studies in some regions of Iran^19, 55, 58^, the most important reason for the difference in these results can be attributed to the type of scenarios used in the modeling. In climate analysis, primarily scenarios focused more on climate change and little on other factors. The report on emission scenarios of the Intergovernmental Panel on Climate Change (IPCC) addressed this shortage by considering both climate and socio-economic changes^59, 60^. In recent years, SSP scenarios have been used as the latest climate models, and not only the greenhouse gas concentration but also how climate change will change in response to socio-economic indicators such as population, economy, land use, and energy change will be considered^61^. This difference may be due to differences in the study area and/or the model used. However, the reference studies did not consider the existence of a correlation between climatic layers and used 19 layers in their study. Furthermore, this could be the result of different global climate models (GCMs) that create specific differences in regional climate change prediction. Therefore, we used SSP-MIROC6 in our study, where as those studies were based on RCP-BCC-CEM2-MR. Apart from climate, other factors such as food conditions, topography, and vegetation conditions can affect the distribution of rodents, so they have stricter criteria than sandflies to choose their habitat^62^. Considering all factors affecting the distribution of species in modeling studies is a major challenge for such studies^33, 63^.

Our results added more detail about suitable areas under future conditions and allowed us to predict potential changes in the future by presenting gain and loss area ranges. According to all scenarios of the 2030s and the 2050s, the MaxEnt model estimated the loss area ​​as more than the gain area for *Ph. sergenti*, *R. opimus*, and *M. libycus* species. The loss areas of these species are mainly related to the central and southern regions of the country and the distribution pattern of these three species is mainly formed by Bio1. Therefore, the annual temperature change probably has a significant effect on the distribution area. This trend observed in our study is consistent with previous findings obtained by similar methods for *R. opimus* species in Iran and the world^18^. Under future conditions, the most stable areas are for *Ph. papatasi*, *T. indica*, and *M. hurrianae* which will probably be less affected by climate change than the rest of the species in this study. Therefore, in the future, these three species may remain a major vector and/or reservoirs in areas that are present under current conditions.

There are several limitations in this study such as the lack of access to NDVI data for the 2030s and the 2050s, therefore, the model interpretation in this study was only based on bioclimatic and topographic variables and did not consider other factors. Despite this, there is no doubt that these models cannot predict the complexity of the real world. However, to better understand this complexity, machine learning-based models such as those we have used here are vital tools in many areas of entomology and VBDs. We suggest that future studies in the field of the distribution of vectors and reservoirs of different diseases should test and evaluate different models in a certain sitting. Furthermore, we can make much more progress in understanding the best performance of a given model by comparing different settings. On the other hand, this study was carried out according to different socio-economic scenarios at the country scale, so our predictions cannot show the characteristics of specific places in Iran at the local scale. We suggest increasing the number of local studies and incorporating some dynamic parameters (such as growth rate, species migration ability, competitive interactions, or species sensitivity to climate). It is also important to be aware of the seasonal changes in vector activity. Therefore, our last recommendation is to more accurately determine the CL pattern in the country. In addition to the spatial changes, the temporal changes of the vectors should also be predicted.

## Conclusion

Over the past few years, there has been a growing body of evidence that climate change has occurred more frequently than ever before. Although attributing the changing pattern of VBDs to any other factor cannot be ruled out, most researchers do not doubt that climate change also plays an important role. It seems that there is a knowledge gap in applying climatic scenarios to predict the risks of various VBDs in Iran. To fill this gap, the use of a set of SSP scenarios in our study provided a more complete understanding of how climate and socio-economic indicators can interact to impact the shifting range of CL vectors and reservoirs in Iran by 2050. We expanded upon previous research by providing more detail about areas suitable for the distribution of CL vectors and reservoirs under climate change conditions, and by presenting gain and loss area ranges. We highlight that the use of different modeling techniques is beneficial to predict the potential distribution of vectors and reservoirs, which can help to reduce the burden of VBDs, especially in vulnerable areas. However, it is essential to consider that the performance of a particular model may vary depending on several factors such as sample size, environmental variables selected, and spatial scale. Furthermore, we also emphasize addressing the impact of climate change on VBDs transmission in Iran and call for more studies to assess the impact of climate change on the epidemiology of VBDs. Our main findings showed that potential changes in the distribution of CL vectors and reservoirs across the country, and the risk of CL transmission at a country level, which can be valuable scientific evidence for CL management in the pre-emergency phase. It may help compile threat control strategies, improved healthcare, and economic systems should be established in advance to better respond to potential global risks and their long-term severe impacts in the future.

## Material and methods

### Occurrence data

**Figure 6:**
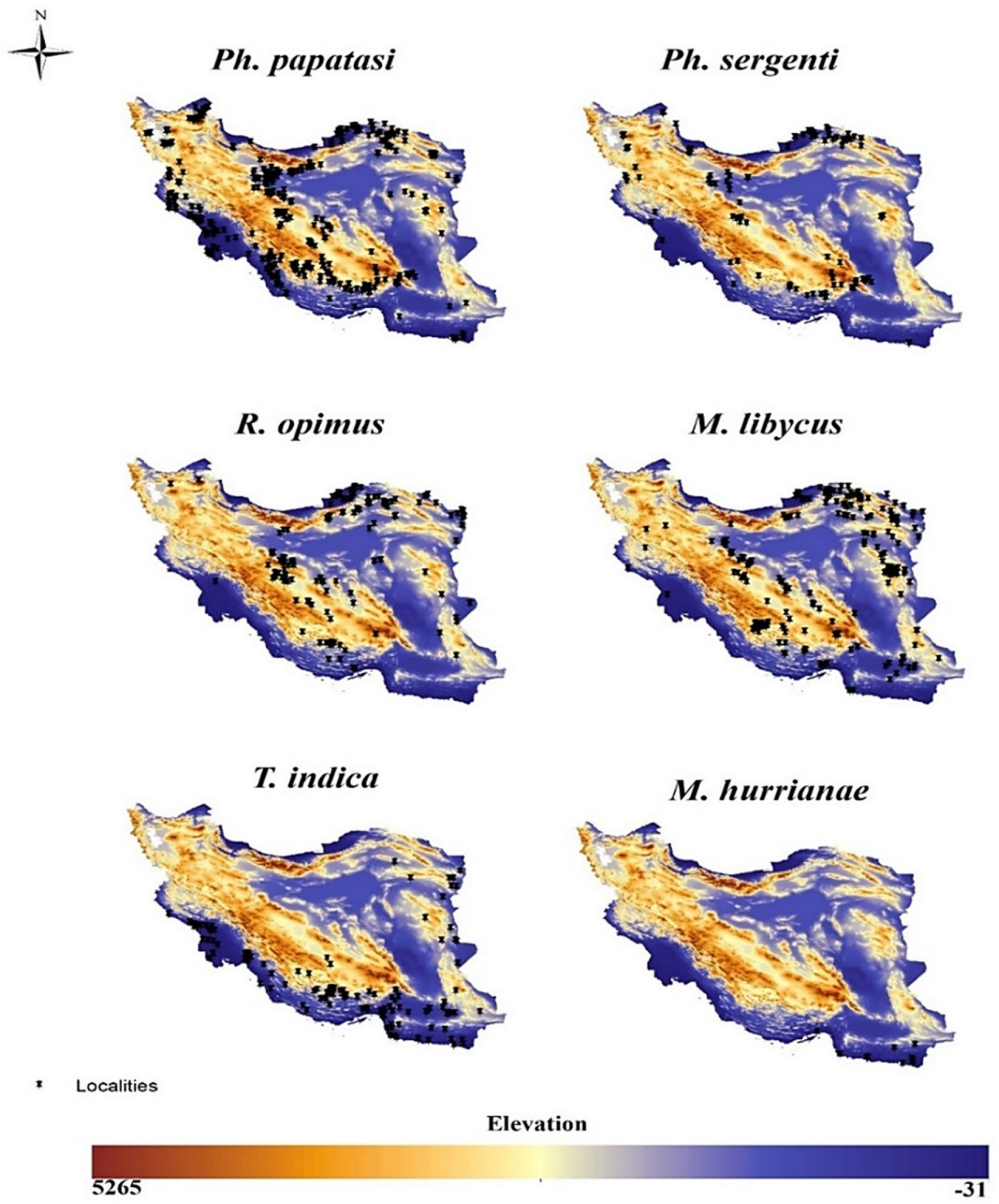
Distribution maps of the two vectors (*Phlebotomus papatasi* and *Phlebotomus sergenti*) and the four reservoirs (*Rhombomys opimus*,* Meriones libycus*,* Tatera indica*, and *Meriones hurrianae*) of cutaneous leishmaniasis in Iran.

For the creation of the Excel database of two vectors (*Ph. papatasi*, *Ph. sergenti*) and four reservoirs (*Rh. opimus*, *M. libycus*, *T. indica*, *M. hurrianae*) of CL, we surveyed their presence points in Iran during the years 2011–2021, through different online scientific sources (Google Scholar, PubMed, Web of Science, SID, Irandoc, Magiran). Geographic coordinates of species were gathered using keywords related to the name of species. Considering that the occurrence records were collected from different data sources we first removed duplicates. Then to avoid pseudo-replication and spatial autocorrelation we removed distribution points that were close to each other (distance ≤ 1 km) for each vector and reservoir separately. Data were cleaned and duplicated and those at distances < 1 km were removed using a spatially rarefy occurrence data tool in SDMs toolbox v2.5 (ArcGIS 10.5 software)^64^. Database imported into ArcMap 10.5 software and displayed on the map (Fig. 6).

### Environment data

Current (1970–2000) and future (2020–2040 and 2041–2060) bioclimatic data collected on WorldClim (v2.1.) (www.worldclim.org) with 1 km^2^ resolution. For both future periods, a couple of SSP (1–2.6 and 5–8.5) from the sixth version of the Model for Interdisciplinary Research on Climate (MIROC6) were chosen to represent low and high-concentration emission scenarios of greenhouse gases, respectively, and were used to predict the future distribution of CL vectors and reservoirs under different climatic changes scenarios. In addition, the altitude layer was also downloaded with a spatial resolution of 1 km^2^ from the WorldClim (v2.1.) website. To prepare layers, we imported bioclimatic/topography (raster format) layers in ArcGIS 10.5 and clip using the Iran boundary shape file.

Pearson correlation test for "bioclimate" and "topography" layers (sdm toolkit) by ArcGIS v10.5 excluding highly correlated layers (r > 0.7). Of the 20 available layers (19 bioclimatic layers and one altitude layer) seven variables were retained for modelling^65^ (Table 4).

**Table 4:** Bioclimatic variables used in species distribution modeling.

Abbreviations	Variables
BIO1	Annual mean temperature (°C)
BIO 2	Mean diurnal range: mean of monthly (max temp–min temp; °C)
BIO 3	Isothermality: (Bio2/Bio7) × 100
BIO 4	Temperature seasonality (standard deviation × 100)
BIO 12	Annual precipitation (mm)
BIO 15	Precipitation seasonality (coefficient of variation)
ALT	Altitude (m)

### Climate modeling

MaxEnt^66^ and RF^67^ modeling techniques were used to predict the impacts of climate change on the change in the distribution of CL vectors and reservoirs in Iran using MaxEnt v3.4.3 software^66^ and “sdm” package^68^ in R environment 4.1.3. We used customized settings for modeling; therefore, we chose 10,000 pseudo-absences as the maximum number of background points for both algorithms. Furthermore, to assess the model’s performance 80% of the occurrence data were used for training (model calibration), whereas the remaining 20% were used for testing (model evaluation) with 10 repetitions.

### Evaluation of model performance

This was performed using the area under the curve (AUC) metric independent of the threshold. This approach is one of the most frequently used in statistics for model evaluation in niche modeling studies^43, 69^. The AUC measures the predictive performance of models with values between 0 and 1. According to the AUC criterion, a value equal to 0.5 represents a model without good predictive power (indicating random prediction). Models with an AUC greater than 0.75 are acceptable, and models with an AUC greater than 0.9 are considered excellent, simply stated the higher AUC value shows the higher performance of the model^70^. To represent changes in suitable habitat, we summarized the results of distributional shifts in the ranges of species qualitatively and quantitatively among the two models per SSP scenario. Specifically, we mapped stable, gain, and loss areas in species distribution under future climate change scenarios.

### Field validation of the distribution model

**Figure 7:**
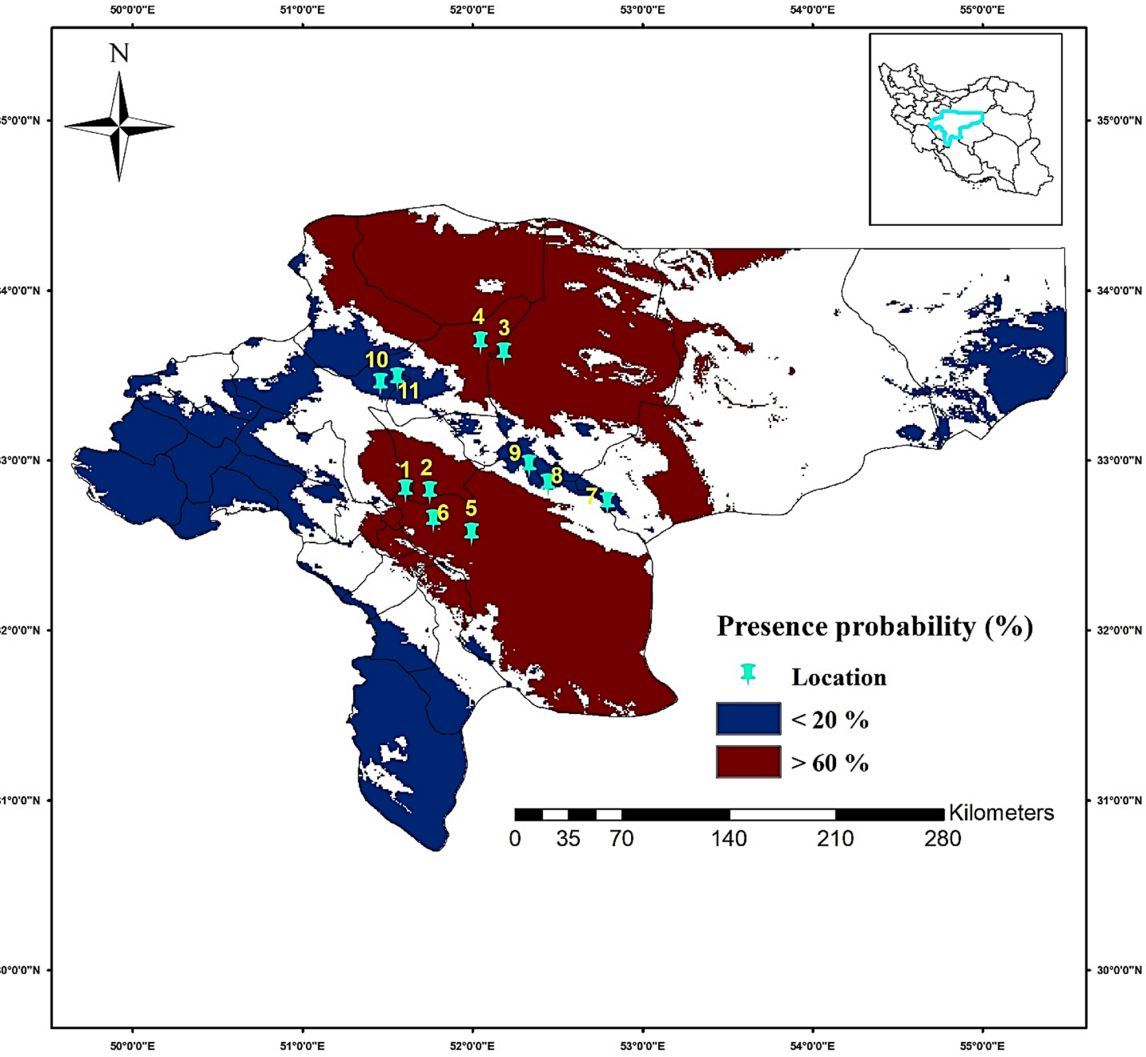
The location of the selected districts to collect *Phlebotomus papatasi*, *Rhombomys opimus*, and *Meriones libycus* species with presence probability < 60 and > 20% in Esfahan Province, Iran.

Esfahan province was chosen because it is known as the most important focus of CL in Iran. Current distribution pattern for *Ph. papatasi*, *R. opimus* and *M. libycus*^5^. Esfahan is located in the center of the country and covers an area of approximately 107,000 km^2^ between 30° 42′ and 34° 30′ N latitude and 49° 36′ and 55° 32′ E longitude (Fig. 7). We selected the current distribution models of *Ph. papatasi*, *R. opimus*, and *M. libycus* which have the widest distribution range among the six studied species in Esfahan Province. Eleven districts from five counties in Esfahan Province were randomly selected. Six districts represent the presence areas of three species with a probability of over 60% and five districts represent the presence areas of the same three species with a probability of less than 20% (Fig. 7, Table 3). It should be noted that to avoid sampling bias in the evaluation of the models, we selected areas where no studies have been conducted regarding the presence of these species until then ^personal communication^. Specimens were collected from rodent burrows and near their breeding places during July 2022, the month when *Phlebotominae* sand flies in Esfahan showed the highest activity^71^, using the usual methods of collecting the sand flies (sticky traps) and rodents (Sherman live traps) (Supplementary material: Figs. S1–S3). Sampling was performed once in each area using 40 sticky traps and 30 Sherman traps. In total, 440 sticky and 330 Sherman traps were installed in the 11 districts. To identify the specimens, the traps were collected and transported to the laboratory of Esfahan Health Research Station, Tehran University of Medical Sciences. *Phlebotominae* sand fly specimens were dissected on a glass slide, the entire head was separated with a needle and mounted in Puri’s medium for later identification. The sand flies were identified based on morphometric characters^72^ under a light microscope (Supplementary material: Fig. S4). Rodents were also identified based on their morphological characteristics (Supplementary material: Fig. S5)^73^.

### Ethical approval

This study was conducted under the ethical principles, national norms and standards for conducting Medical Research in Iran. The Research Ethics Committees of the School of Public Health & Allied Medical Sciences-Tehran University of Medical Sciences approved this project under code: IR.TUMS.SPH.REC.1400.107.

## Supplementary Information

Supplementary Information.

## Data Availability

All data needed to evaluate the conclusions in the paper are present in the paper and/or the Supplementary Materials, or the references cited here within.
