# Supplementary material for: Predicting current and future high-risk areas for vectors and reservoirs of cutaneous leishmaniasis in Iran
Source: Sci Rep. 2023 Jul 17;13:11546. doi: 10.1038/s41598-023-38515-w (PMC10352301; doi:10.1038/s41598-023-38515-w)
Supplement: Supplementary file 1 — Supplementary Information. [file 41598_2023_38515_MOESM1_ESM.pdf]

Supplementary Information

Predicting current and future high-risk areas for vectors and reservoirs of cutaneous leishmaniasis in Iran

Faramarz Bozorg-Omid, Anooshe Kafash, Reza Jafari, Amir Ahmad Akhavan, Mohammad Rahimi, Abbas Rahimi Foroushani, Fahimeh Youssefi, Mohammad Reza Shirzadi, Abbas Ostadtaghizadeh, Ahmad Ali Hanafi-Bojd

Figures S1–S

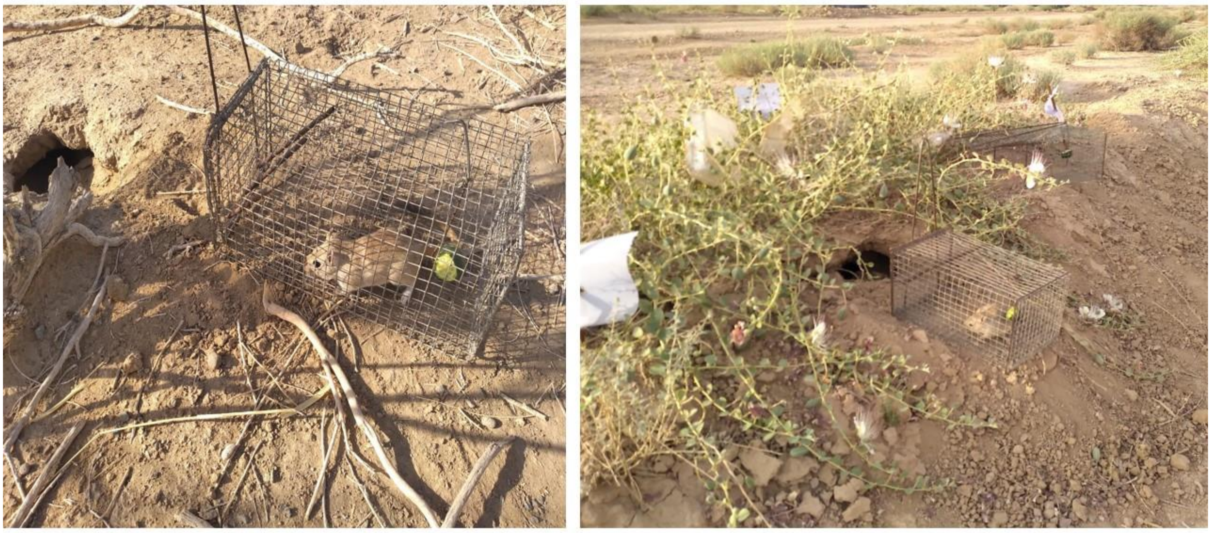

Supplementary Fig S1. Sherman live traps. Rodents collection sites from Fassaran district, Esfahan Province of Iran, 2022.

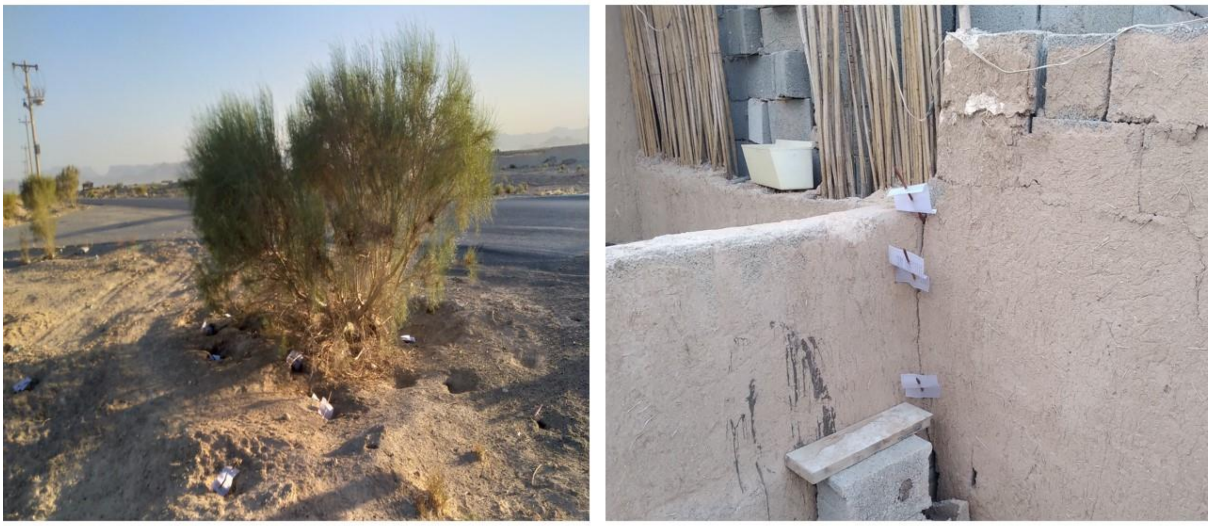

Supplementary Fig S2. Sticky traps. Sand flies collection sites from Fassaran district, Esfahan Province of Iran, 2022.

.

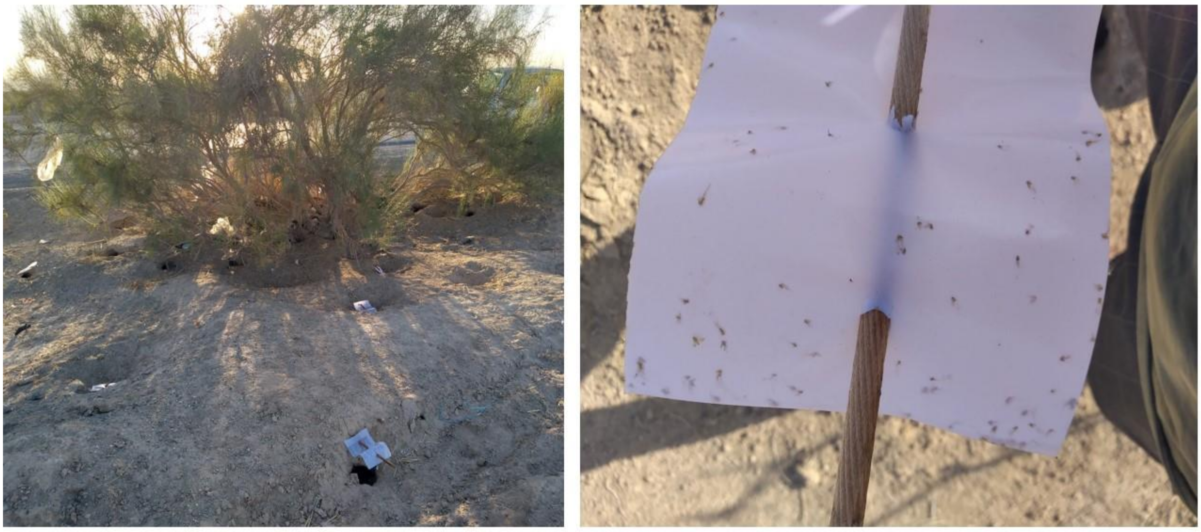

Supplementary Fig S3. Sticky traps. Sand flies collection sites from Fassaran district, Esfahan Province of Iran, 2022.

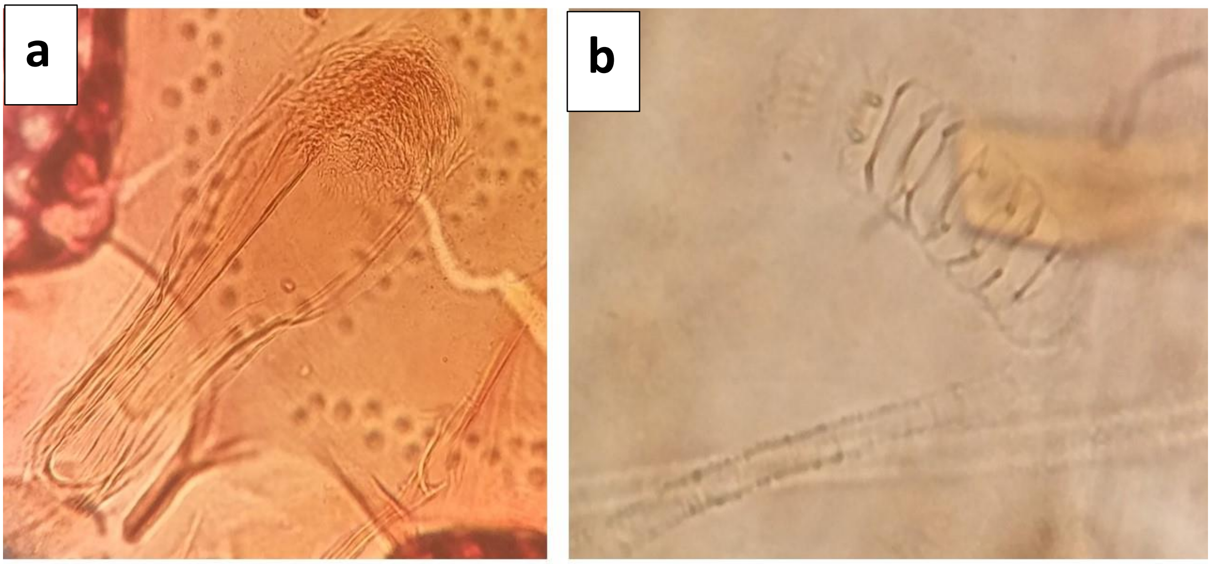

Supplementary Fig S4. Identification based on morphometric characters. Morphological details of the a) pharynx and b) spermathecae for the female *Phlebotomus papatasi*.

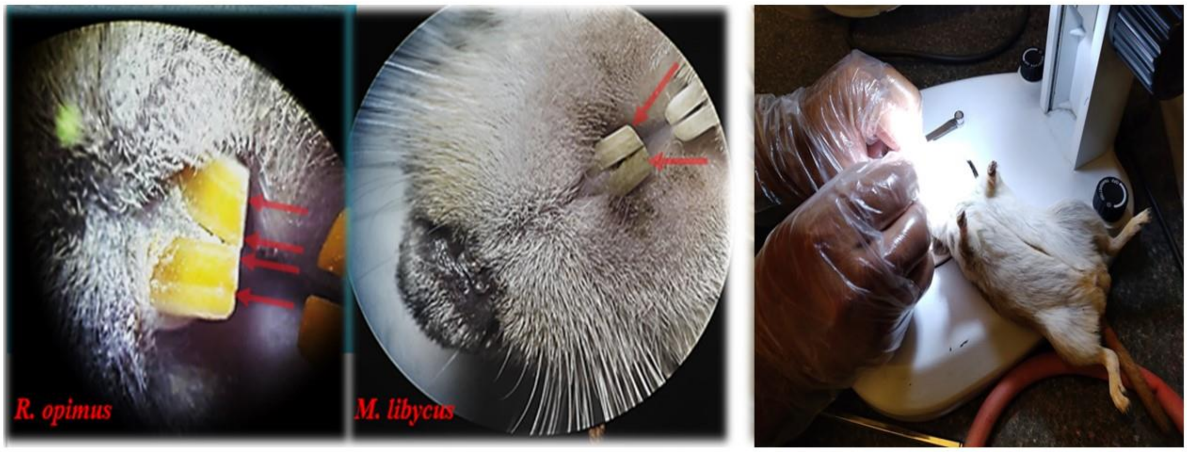

Supplementary Fig S5. Identification based on tooth morphometric characters. Morphological details of the teeth for *Rhombomys opimus* and *Meriones libycus*.
